# Perception of the Rural Community Regarding the Role of Nursing Professionals: A Study in the High Andean Regions of Peru

**DOI:** 10.3390/nursrep15050148

**Published:** 2025-04-29

**Authors:** Nelly Martha Rocha Zapana, Elsa Gabriela Maquera Bernedo, William Harold Mamani Zapana, Angela Rosario Esteves Villanueva, Nury Gloria Ramos Calisaya

**Affiliations:** Facultad de Enfermería, Universidad Nacional del Altiplano, Puno 21001, Peru; nmrocha@unap.edu.pe (N.M.R.Z.); egmaquera@unap.edu.pe (E.G.M.B.); wmamani@unap.edu.pe (W.H.M.Z.); aresteves@unap.edu.pe (A.R.E.V.)

**Keywords:** rural population, community health nursing, community health services, health services accessibility

## Abstract

Access to healthcare services in rural areas of Peru remains a challenge, with marked differences compared to urban areas. Despite the importance of primary healthcare (PHC) in these communities, the rural population has a negative perception of the role of nursing staff. This study aimed to assess the perceptions of residents in the highland communities of Huata and Ichu, Province of Puno, located in southern Peru, regarding the role of nursing professionals. The general perception of the residents was first analyzed, followed by an evaluation of two dimensions (Fieldwork and Health Education), which allowed for the development of the REFCO (Role of the Nursing professional in the community) scale. The sample included 329 rural adults, mainly between 30 and 59, predominantly female, with incomplete secondary education. The results showed an unfavorable perception (54%) of nursing staff performance. The fieldwork dimension reported low visibility of nursing activities in the community, such as home visits and health programs. The second dimension demonstrated that educational interventions were perceived as infrequent and of low impact, with insufficient adaptation to local needs. These results highlight the need to strengthen the presence and educational strategies of nursing staff in alignment with the cultural and demographic realities of rural communities. Furthermore, it suggests a greater need for interinstitutional collaboration and a more personalized approach to community activities to improve the perception and effectiveness of healthcare services in these areas.

## 1. Introduction

In Peru, the total population is approximately 32 million, with 79% living in urban areas and 21% in rural areas [1,2]. Both populations require access to healthcare services to ensure their well-being and quality of life and to prevent or treat specific diseases [3]. Although 90% of the urban population has access to healthcare, only 60% of the rural population has this benefit [4]. This disparity is due to several factors, including inadequate infrastructure in rural areas, a shortage of healthcare personnel, and transportation challenges.

Although urban residents may have access to specialized healthcare services, the rural population only has access to primary healthcare services. Primary healthcare (PHC) is a model designed to be accessible to rural families [5]. It focuses on disease prevention, basic diagnosis and treatment, maternal and child health, basic mental health services, and rehabilitation [6,7]. In general, an interdisciplinary team of professionals, including general practitioners, nurses, midwives, nursing technicians, and nutritionists, generally provides this care to rural populations [8].

Nursing professionals have a crucial role in PHC, ensuring comprehensive and quality care in rural communities. Their work promotes health and disease prevention while providing continuous and direct care to vulnerable populations [9,10]. However, rural communities often perceive the healthcare professionals provide as inadequate, likely due to cultural and linguistic differences, limited resources, and a preference for traditional medicine over conventional healthcare [11].

Rijcharismo was a health movement that aimed to promote collaboration between healthcare professionals and rural communities through the establishment of health brigades and health education activities [12]. This approach fostered a positive perception among rural populations by showcasing healthcare professionals’ commitment to understanding and respecting local culture and needs, increasing trust and appreciation for the role of nursing in community health [13]. Thus, developing strategies for evaluating nursing staff performance is essential to implement interventions that enhance healthcare services for rural populations.

Different evaluation scales like Role of the Nursing professional in the community (REFCO), 360°, and Competencies have been used to assess healthcare personnel. These scales measure job performance, identify strengths, and facilitate professional development and service quality [14,15,16,17]. In particular, the REFCO scale is well-suited for healthcare settings due to its specific design. It measures technical and behavioral skills crucial in healthcare environments, providing precise and actionable feedback and promoting continuous improvement among healthcare staff [15]. Thus, the methodological application of this technique will allow concrete evaluation data on nursing staff performance and provide feedback on their work.

Under these considerations, the objective of this study was to evaluate the perceptions of rural residents in the highland communities of the districts of Huata and Ichu, which are located in southern Peru, regarding the role of nursing professionals.

## 2. Materials and Methods

The study primarily utilized a quantitative, cross-sectional survey design to assess the perceptions of rural residents toward nursing professionals. The main element of the study involved administering the REFCO scale to a sample of 329 participants. Although the main findings presented in this paper are based on the quantitative analysis of the survey data, the results provided valuable contextual information on the role of nursing professionals.

**Figure 1:**
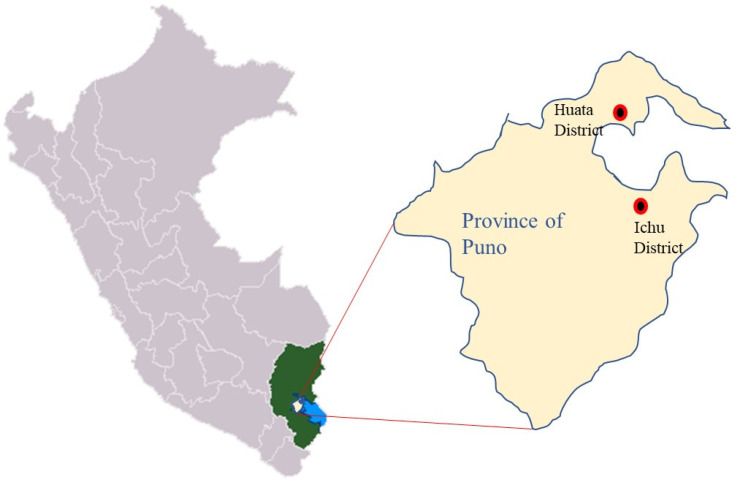
Geographic location of the districts of Huata and Ichu, Province of Puno, Department of Puno, Peru.

This study was conducted in communities within the districts of Huata and Ichu, located in the Province of Puno, Department of Puno, Peru. These areas were chosen due to their unique demographic characteristics, including an altitude (between 3811 and 3850 m above sea level), as well as populations with Quechua and Aymara ancestry (Figure 1).

A census-based sampling method was employed to establish the study population. The inclusion criteria specified that participants must be the head of the household. The age range of 18 to 59 years was selected, as this typically represents the economically active adult population. Literacy was a requirement for participation. Exclusion criteria included individuals who were not the head of the household, those outside the age range of 18 to 59 years, and individuals who did not reside in the specified communities. As a result, 329 adults were selected for the study, with 199 adults from the community of Collana in the Huata district and 130 adults from the communities of Tunuhuire Grande, Tunuhuire Chico, and Vallecito in the Ichu district.

### 2.1. Instrument for Measuring the Role of the Nursing Professional in the Community (REFCO)

The REFCO scale is a structured instrument in the form of a questionnaire, with items that evaluate the performance and functions of nursing professionals across two specific dimensions: fieldwork and education [14]. These dimensions are detailed as follows:

Dimension: Fieldwork

Q1: Have you observed the nurse participating in creating favorable environments (at home, in schools, in municipalities) within the community?

Q2: Have you observed the nurse participating in health programs (vaccination, educational health sessions, campaigns to prevent sexually transmitted diseases, healthy schools) in the community?

Q3: Have you observed the nurse participating in follow-up, control, and monitoring activities for individuals, families, and the community?

Q4: Have you observed the nurse visiting the homes of community/neighborhood members?

Q5: Have you observed the nurse managing health development projects (family baskets, participatory budgeting) to benefit the community?

Dimension: Education

Q6: Have you observed the nurse providing educational sessions (lectures) either at home, in health centers, schools, or workplaces?

Q7: Have you observed the nurse using audiovisual aids (flip charts, brochures, infographics, videos) and practical demonstrations during educational sessions?

Q8: Have you observed the nurse delivering educational sessions tailored to the needs of individuals, families, and the community?

Q9: Have you observed that the educational sessions provided by the nurse have led to changes in individuals, families, and the community?

This 9-item questionnaire was rated using a five-point Likert scale: Never (1), Rarely (2), Sometimes (3), Almost always (4), and Always (5) [18].

### 2.2. Interview Procedure and Data Collection

Before data collection, members of the research team thoroughly explained the study’s objectives, risks, and benefits to all participants before they signed an informed consent form. In addition, the confidentiality of the data was ensured through the anonymity of the records, and the collected information will be securely stored for five years in accordance with ethical guidelines and institutional policies, and will be available for potential queries or audits. This study received approval from the Ethics Committee of the National University of Altiplano-Puno (CONSTANCIA N°021-2023/CIEI UNA-Puno), in accordance with the established ethical guidelines.

Each participant’s interview lasted 15 to 20 min. During the application process, all questions raised by the participants were addressed, ensuring a proper understanding of each item on the REFCO scale.

### 2.3. Data Analysis

The data collected from the surveys completed by the 329 participants were exported to Microsoft Excel. Then, SPSS Statistics for Windows, Version 28.0 (IBM Corp., Armonk, NY, USA) was used to calculate absolute frequencies, percentages, means, and standard deviations. A chi-square statistical analysis was conducted to assess the association between the categorical variables of the perception of the nurse’s role in the two analyzed dimensions (Health Education and Fieldwork) using Statgraphics Plus for Windows 4.0 (Statpoint Technologies, Inc., Warrenton, VA, USA).

Principal Component Analysis (PCA) was conducted to assess how the categorized variables are related to the population’s perception of the role of nursing staff. The analysis used the Pearson correlation matrix, treating the experimental combinations as individuals and the polyphenol compounds as active variables, while their respective families were considered supplementary variables. The analyses were performed using R software version 4.3.2 (R core Team, 2024), specifically leveraging the FactoMineR package to identify meaningful patterns and associations within the dataset.

## 3. Results

### 3.1. Sociodemographic Description

The target population (n = 329) was primarily female (>54%). The majority of participants were adults aged between 30 and 59 years (>74%). The most common level of education attained was completed secondary school (~37%). The main activities of the population were centered on agriculture, livestock, and domestic work (Table 1).

When examining the distribution by residential sectors, there was a higher concentration of individuals in the district of Huata (~60%) compared to the district of Ichu (~40%). Furthermore, the length of residence among the inhabitants indicates a strong sense of community attachment, with many having spent over 30 years living in the area (Table 1).

### 3.2. Population Perception on the Role of the Nursing Professional

The results indicate that residents of the Huata and Ichu districts generally have a negative perception of nurses. Specifically, 54.1% of respondents rated the nurse’s role as unfavorable, while 44.1% considered it moderately favorable, and only 1.8% viewed it as favorable (Table 2).

### 3.3. Description of the Perception of the Residents Through Two Dimensions (Fieldwork and Health Education)

**Figure 2:**
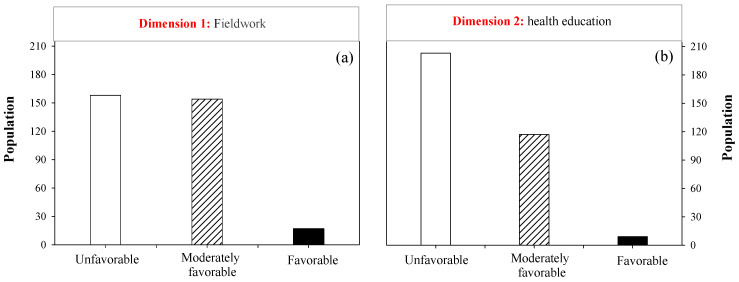
Residents’ perception of nursing staff activities: Fieldwork (**a**) and health education (**b**).

Two key dimensions—fieldwork and health education—were analyzed, which are directly related to nurses’ roles in Huata and Ichu communities (Figure 2). The fieldwork dimension assessed nurses’ active community involvement, accessibility, and ability to take action beyond clinical settings. In contrast, the health education dimension evaluated their effectiveness in empowering the population through knowledge of disease prevention and promotion of healthy habits.

The results indicate a predominantly unfavorable perception of the nurse’s role for both evaluated dimensions. For fieldwork, 48.0% of residents expressed an unfavorable perception, 46.8% showed a moderately favorable opinion, and only 5.2% rated this dimension as favorable (Figure 2a). Interestingly, health education showed a higher negative perception, with 61.7% of responses classified as unfavorable, 35.6% as moderately favorable, and only 2.7% as favorable (Figure 2b).

#### 3.3.1. Description of Gender Perception (Fieldwork)

**Figure 3:**
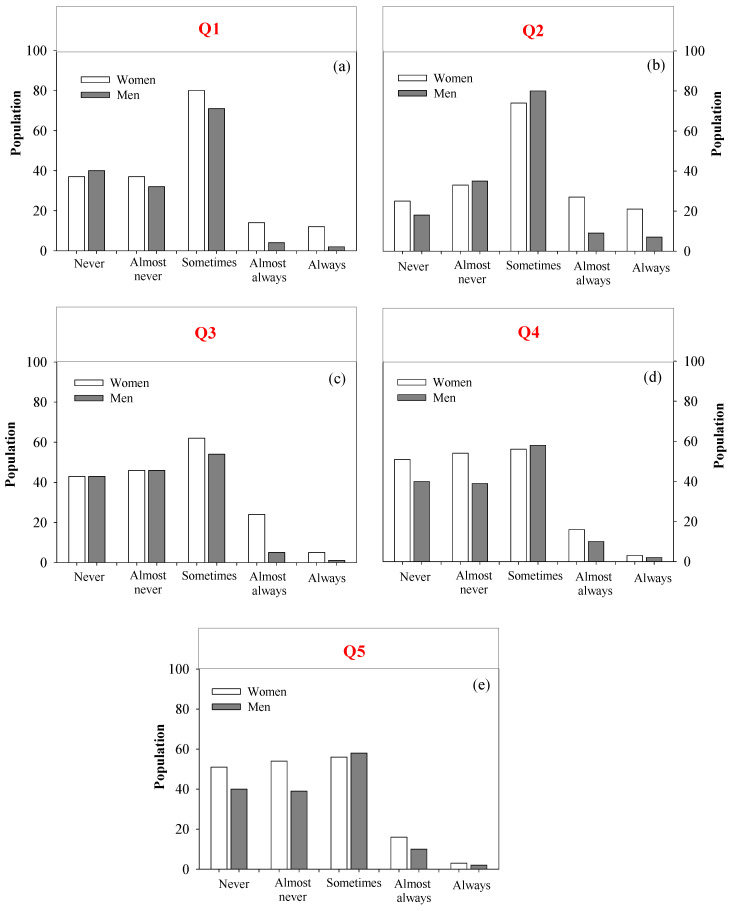
Frequency of Observation of the Activities of the Nursing Professional: (**a**–**e**) represent the questions asked on the REFCO scale.

The majority of people between men and women (45%) believe that nursing staff “sometimes” create a favorable environment (Figure 3a). For gender differences, a slightly higher percentage of women (24%) feel that nurses sometimes foster a positive environment compared to men (21%). The categories for “Never” and “Almost never” reflect relatively low percentages, indicating that most individuals do not hold a completely negative view. However, the percentages for “Almost always” and “Always” are also low, suggesting that there is a need for improvements in these areas.

Similar to Q1, most of the population perceives that nurses “sometimes” participate in health programs. In particular, men have a significantly higher perception (24%) of this “sometimes” category compared to women (20%) (Figure 3b). In contrast, the categories “Never” and “Almost never” have low ratings with 13% and 19%, respectively, indicating that most people do not believe that nurses ever engage in these activities. Additionally, the categories “Almost always” and “Always” also receive low scores, suggesting that the participation of nursing staff in health programs is not very evident to the public.

In Q3, the perception among both men (16%) and women (19%) is that nurses “sometimes” engage in monitoring and control activities (see Figure 3c). Additionally, a higher portion of people believe that nurses either “never” (26%) or “almost never” (28%) participate in these activities. This indicates that the overall population tends to think that nurses rarely engage in monitoring and control activities.

In Q4, the perception remains similar to Q3, where the people consider that nurses “sometimes” make home visits. Although the “Sometimes” category has the highest percentage, there are also significant percentages in the “Never” and “Almost Never” categories. This indicates that the public tends to have a negative view of nurses’ performance in conducting home visits. Finally, in relation to the development of project management, both men and women reported particularly concerning responses, with 77.8% of respondents expressing an unfavorable perception, while only 6.7% provided a positive assessment (Figure 3e).

#### 3.3.2. Description of Gender Perception (Health Education)

**Figure 4:**
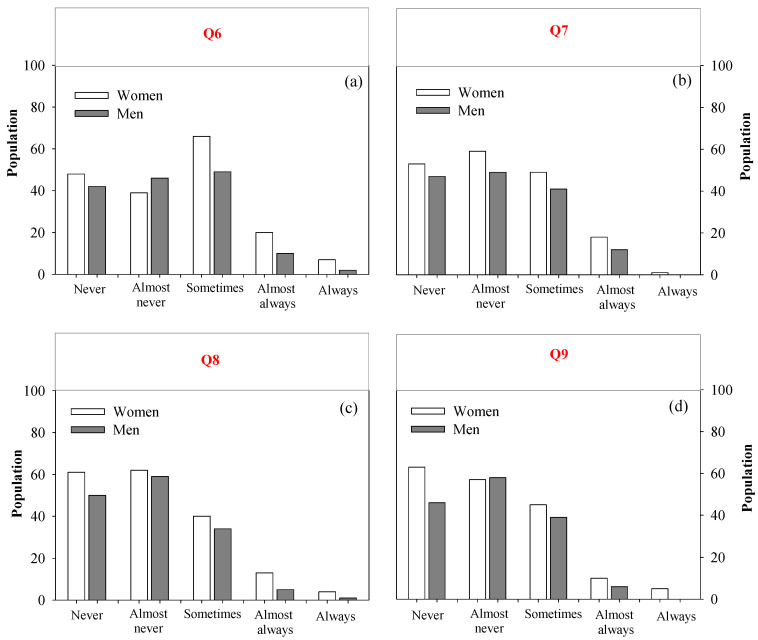
Frequency of Observation of the Activities of the Nursing Professional in the Health Education Dimension: (**a**–**d**) represent the questions asked on the REFCO scale.

The analysis shows that the population views the educational role of nurses as infrequent and having a limited impact (Figure 4). In response to Q6, both men (20%) and women (15%) felt that nurses “sometimes” offer educational sessions. However, it is notable that the “Never” and “Almost Never” categories accounted for 53%, indicating a generally negative perception among the population (Figure 4a). The results for Q7 indicate that 33% of people believe nursing staff “almost never” use audiovisual resources (Figure 4b). This perception is more pronounced among women (59%) compared to men (47%). The low rates of participation in the “Almost always” and “Always” categories highlights an opportunity to enhance communication and visibility regarding these activities.

On the other hand, Q8 and Q9 show that the adaptation of educational sessions to meet specific needs and their impact on the community are viewed as inadequate (Figure 4c,d). Over 70% of respondents believe that these activities are seldom tailored to the population’s needs and that the resulting changes are minimal.

### 3.4. Multivariate Analysis of the Perception of the Role of the Nursing Professional

**Figure 5:**
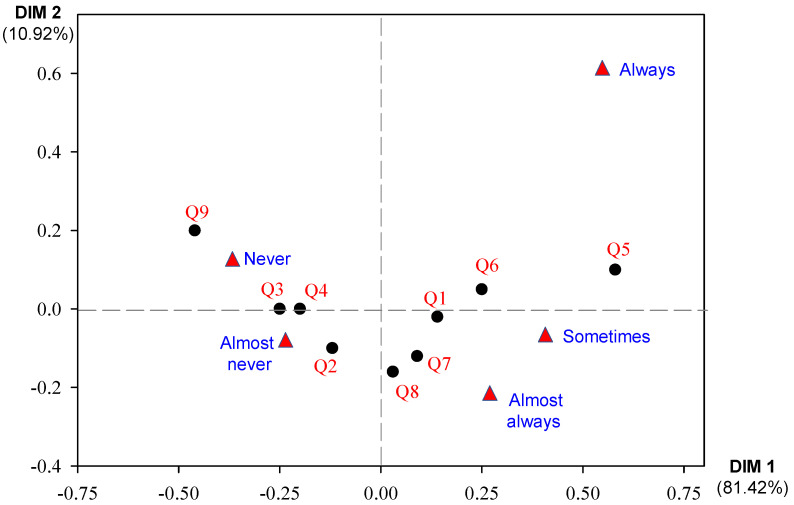
Multivariate analysis of the perception of the role of nursing staff. Q1, Q2, Q3, Q4, Q5, Q6, Q7, Q8, and Q9 are the variables classified in the methodology as part of the REFCO scale.

The figure shows a principal components analysis (PCA) of responses to nine questions (Q1–Q9) regarding perceptions of the nursing staff’s role. PCA is a statistical method that reduces the complexity of data by identifying the underlying variables (principal components) that account for most of the variance in the original dataset. In this sense, dimension 1 (DIM 1) and dimension 2 (DIM 2) explain 92.34% of the total variance, with DIM 1 accounting for 81.42% and DIM 2 accounting for 10.92% (Figure 5).

Questions Q5 and Q6 are situated in the upper right quadrant, indicating that they are associated with high scores on both principal components. This suggests that these questions pertain to perceptions related to nursing staff’s project development and health education. Conversely, questions Q2 and Q8 are found in the lower left quadrant, which indicates low scores on both principal components. These questions relate to perceptions of health program implementation and community educational sessions, respectively.

Questions Q3, Q4, and Q9 are located in the upper left quadrant, which suggests that they are associated with high scores on DIM 2 and low scores on DIM 1. These questions address perceptions of follow-up and monitoring activities, home visits, and the impact of educational sessions, respectively. Notably, question Q1 is positioned close to the origin, indicating that it is not strongly associated with either of the two principal components. This question pertains to an aspect of the nursing staff’s role that is not well captured by the two principal components.

## 4. Discussion

The studied population reflects typical demographic features of the high Andean regions of Peru, where individuals are primarily involved in agricultural and livestock activities, with a notable predominance of females. Acosta-Salazar et al. [19] indicate that rural communities often have strong cultural roots and a reliance on traditional economic activities. Additionally, the low prevalence of completed secondary education (approximately 37%) and higher education (around 7%) highlights the limited progress in educational access. This situation may affect the understanding and appreciation of the role of nursing staff.

According to our results, residents hold an unfavorable perception (54.1%) of the role of nursing professionals. This negative perception is further underscored by the evaluation of two key dimensions: fieldwork and health education. Specifically, residents reported low visibility of nursing activities in the community setting like home visits and participation in health programs. Furthermore, educational interventions were perceived as infrequent, lacking impact, and not sufficiently tailored to the cultural and demographic realities of these rural communities, indicating a significant area for improvement in the presence and effectiveness of nursing staff.

Research from other countries has demonstrated that specific cultural beliefs and practices can impact communication styles and health understanding, which in turn hinders the integration and recognition of nursing professionals [20,21]. In Peru, the Rijcharismo program was introduced to enhance community perceptions of healthcare professionals by actively incorporating local traditions into healthcare practices [12]. Despite such initiatives, negative perceptions of nursing staff may still persist. This can be attributed to several factors, including the perception that nurses’ roles are secondary to those of other health professions, such as doctors, as well as cultural and communication barriers that obscure the recognition of nursing work [22,23]. To address these issues, it is essential to analyze specific dimensions that contribute to this negative perception, identifying the factors or activities that play a significant role.

The negative results observed in fieldwork and health education indicate deficiencies in the direct interaction between nurses and the community, as well as shortcomings in implementing effective educational strategies (Figure 1). Home visits and direct interaction are crucial for strengthening the relationship between healthcare professionals and residents, particularly in rural areas where personalized care fosters trust and enhances perceptions of service quality [24,25]. Thus, it is likely that there needs to be more structured community activities, a lack of which may lead to the perception of the nurse’s role as peripheral or insignificant [26].

In relation to the health education dimension, the negative perception may indicate that educational strategies are not being tailored to the cultural and linguistic realities of the community [9]. Thus, it is essential to implement a tool that measures the role of nursing staff in the community using specific variables related to the community [14,27].

Our results indicate that residents have a limited understanding of the nurse’s role in creating supportive environments, participating in health programs, conducting follow-up activities, making home visits, and managing development projects. Previous studies have demonstrated that nurses’ involvement in creating favorable environments and participating in health programs directly influences the prevention of chronic diseases and encourages healthy lifestyle habits [28]. However, despite the potential for nursing staff to contribute to health promotion, their involvement is often restricted by a lack of collaboration with local governments [29]. Additionally, Grajales [30] reported that the excessive administrative workload and the high patient-to-nurse ratio hinder nurses’ ability to fully engage in follow-up activities and home visits.

This study benefits from its focus on an understudied rural population, providing valuable insights into their perceptions of nursing professionals, a crucial aspect for improving primary healthcare delivery. However, the cross-sectional design could limit the ability to establish or observe changes over time. The reliance on reported survey data may also introduce potential biases. Additionally, the findings are specific to the communities of Huata and Ichu in the Puno region, which may limit their generalizability to other rural contexts with different cultural and socioeconomic characteristics.

## 5. Conclusions

The study reveals a concerning trend: residents of the Huata and Ichu districts have a negative perception of nursing professionals. This unfavorable view is evident among both men and women, reflected in their overall assessments and evaluations of specific aspects such as fieldwork and health education. It indicates a disconnect between the community’s expectations and the actions of nurses. Several factors may contribute to this negative perception, including the limited visibility of nurses’ activities, cultural and communication barriers, and a potential lack of effective community engagement strategies tailored to the local context. The findings highlight an urgent need for interventions to enhance the visibility of nursing roles, improve communication and cultural sensitivity in service delivery, and strengthen community engagement through more structured fieldwork and health education initiatives.

## Figures and Tables

**Table 1:** Sociodemographic characteristics of the participants within the districts of Huata and Ichu, located in the Province of Puno, Peru.

Descriptions		f	%
Age	18 to 29 years	81	24.6%
	30 to 59 years	244	74.2%
	>60 years	4	1.2%
	Total	329	100.0%
Gender	Female	180	54.7%
	Male	149	45.3%
	Total	329	100.0%
Level of Education	Incomplete primary	43	13.1%
	Complete primary	54	16.4%
	Incomplete secondary	50	15.2%
	Complete secondary	121	36.8%
	Incomplete Superior	38	11.6%
	Complete Superior	23	7.0%
	Total	329	100.0%
Occupation	Farmer	61	18.5%
	Bricklayer	29	8.8%
	Housewife	89	27.1%
	Driver	3	0.9%
	Merchant	33	10.0%
	Student	27	8.2%
	Livestock Farmer	67	20.4%
	Professional	11	3.3%
	Technician	9	2.7%
	Total	329	100.0%
Areas	Huata—1° Collana	197	59.9%
	Ichu—estación	42	12.8%
	Ichu—Checarmaya	6	1.8%
	Ichu—Tunuhuire grande	44	13.4%
	Ichu—Tunuhuire chico	40	12.2%
	Total	329	100.0%
Years of residence	1 to 9 years	50	15.2%
	10 to 19 years	50	15.2%
	20 to 29 years	75	22.8%
	>30 years	154	46.8%
	Total	329	100.0%

f represents the number of people.

**Table 2:** Perception of the professional role of community nursing.

	**f**	**%**
Unfavorable	178	54.1%
Moderately favorable	145	44.1%
Favorable	6	1.8%
Unfavorable	329	100.0%

f represents the number of people.

## Data Availability

The raw data supporting the conclusions of this article will be made available by the authors on request.

## Public Involvement Statement

There was no public involvement in any aspect of this research.

## Guidelines and Standards Statement

This manuscript was prepared following the guidelines of the Ethics Committee at the National University of Altiplano-Puno.
